# Recent Progress in Magnetic Resonance Imaging of the Embryonic and Neonatal Mouse Brain

**DOI:** 10.3389/fnana.2016.00018

**Published:** 2016-03-03

**Authors:** Dan Wu, Jiangyang Zhang

**Affiliations:** ^1^Department of Radiology, Johns Hopkins University School of MedicineBaltimore, MD, USA; ^2^Bernard and Irene Schwartz Center for Biomedical Imaging, Department of Radiology, New York University School of MedicineNew York, NY, USA

**Keywords:** magnetic resonance imaging, microscopy, brain anatomy, brain development, embryonic mouse brain, high resolution

## Abstract

The laboratory mouse has been widely used as a model system to investigate the genetic control mechanisms of mammalian brain development. Magnetic resonance imaging (MRI) is an important tool to characterize changes in brain anatomy in mutant mouse strains and injury progression in mouse models of fetal and neonatal brain injury. Progress in the last decade has enabled us to acquire MRI data with increasing anatomical details from the embryonic and neonatal mouse brain. High-resolution *ex vivo* MRI, especially with advanced diffusion MRI methods, can visualize complex microstructural organizations in the developing mouse brain. *In vivo* MRI of the embryonic mouse brain, which is critical for tracking anatomical changes longitudinally, has become available. Applications of these techniques may lead to further insights into the complex and dynamic processes of brain development.

## Introduction

The mammalian brain undergoes rapid growth during the prenatal and neonatal periods. Structural changes, from the formation of basic functional units and neural circuitry to axonal pruning and myelination, are critical for normal brain functions at the adult stage. In order to investigate normal and pathological changes during these critical periods, advanced imaging tools have been developed to dissect the developing brain from macroscopic (Toga et al., 2012; Van Essen et al., 2013) to microscopic (Helmstaedter et al., 2013; Takemura et al., 2013) levels. Magnetic resonance imaging (MRI) has been increasingly used in the clinics to examine fetal brain development and injuries (Sevely and Manelfe, 2001; Limperopoulos and Clouchoux, 2009). Compared to other clinical imaging modalities that are commonly used to image the developing brain, primarily ultrasound, MRI provides high resolution and rich tissue contrasts for delineation of brain structures as well as several diagnostic markers for detecting fetal brain injuries (Rutherford, 2002). Once abnormalities in the brain are detected by ultrasound, MRI is the technique of choice in the clinic to establish the pattern of injuries (Sevely and Manelfe, 2001). Even though fetal brain MRI is increasingly adopted, there are still many questions remained to be answered. For example, what are the relationships between MRI signals and brain microstructures under normal and pathological conditions in the developing brain? To answer these questions, it is necessary to have model systems that allow direct comparisons between MRI signals and histopathology.

The laboratory mouse has been extensively used to study the genetic control mechanisms of brain development and insult-pathology correlation. Mouse brain development has also been a major focus area of neuroscience research, and a tremendous amount of resources have been generated over the last decade, e.g., the Allen developing mouse brain atlas (http://developingmouse.brain-map.org). With the increasingly sophisticated gene technology (e.g., *in vivo* gene transfer Saito and Nakatsuji, 2001), there is an acute demand for high-throughput and sensitive techniques for screening anatomical phenotypes in genetically modified mouse brains during embryonic and neonatal development. In addition, several mouse models of fetal and neonatal brain disorders (e.g., Vannucci and Vannucci, 2005; Burd et al., 2009, 2010) have been established, and imaging tools that can sensitively detect disease progression in these and similar models will be beneficial for understanding the mechanisms of injury and the development of new treatments. While histology has been commonly used to characterize anatomical phenotypes, MRI has its unique advantages as described previously (Johnson et al., 2002; Turnbull and Mori, 2007). For example, the data are in 3D format and digitized, which is convenient for quantitatively analysis. Without the sectioning and staining processes, which are time consuming and may introduce tissue damages and deformation, MRI can provide whole brain coverage and does not require a prior knowledge of the location of anatomical changes, albeit at much lower resolution and specificity. Furthermore, MRI can potentially be used to acquire longitudinal data to capture the dynamic processes of brain development and to characterize disease progression.

During the last decade, tremendous progresses have been made in using MRI to study mouse brain development as described in several excellent articles. These articles cover early pioneering works on the use of MRI to study vertebrate animal development (Effmann et al., 1988; Smith et al., 1994; Jacobs et al., 1999); the development of high-resolution MRI (or MR microscopy) for virtual dissection of the adult mouse brain (Badea et al., 2009), as well as the developing mouse brain (Petiet et al., 2008); the development of advanced MRI contrasts for structural delineation in the developing mouse brain and comparisons with optical and ultrasound imaging (Turnbull and Mori, 2007), and quantitative characterization of brain development (Verma et al., 2005; Zhang et al., 2005; Baloch et al., 2009; Chuang et al., 2011; Ingalhalikar et al., 2015). For a general review on techniques commonly used for imaging the developing mouse brain, two recent review articles provide rather comprehensive lists with pros and cons (Nieman et al., 2011; Norris et al., 2013b). In this review, we mostly focus on the latest developments in using MRI to study the developing mouse brain.

## *Ex vivo* high-resolution MRI of the developing mouse brain

The average volume of an adult human brain is ~3000 times of that of an average adult mouse brain (Badea et al., 2009). In order to reliably delineate structures in the mouse brain, a spatial resolution of 0.1 mm or higher is often needed. As the size of individual voxel becomes smaller, the signals originated from each voxel decrease proportionally. This reduction in signals can be partially compensated by using high field magnets (7 T or higher), high-sensitivity coils that closely match the brain in terms of size and geometry, the addition of signal enhancing contrast agents, and more signal averages at the cost of prolonged imaging time. Acquiring high-resolution images also demands gradient systems that can generate strong and fast-switching magnetic field gradients for efficient spatial-encoding. In general, it is necessary to have a gradient system capable of generating 400 mT/m or higher gradient strength and fast slew rates in order to reach a spatial resolution of 0.1 mm or higher (Johnson et al., 2002). These conditions can be more easily met in *ex vivo* imaging than *in vivo* imaging, as the brain can be dissected out to fit into the most sensitive coil and imaged for several hours on high field preclinical MR systems with high performance gradient systems. In this section, we reviewed several recent developments in *ex vivo* imaging of the embryonic and neonatal mouse brain.

### *Ex vivo* T_1_/T_2_-weighted MRI of the developing mouse brain

T_1_/T_2_-weighted and diffusion MRI have been commonly used to study the developing mouse brain (Mori et al., 2001; Johnson et al., 2002; Mori and Zhang, 2006; Petiet et al., 2008; Zhang et al., 2012). One commonly used procedure to increase signal-to-noise ratio (SNR) in *ex vivo* high-resolution MRI is the application of Gadolinium (Gd)-based contrast agents, e.g., Gd-DTPA. It has been shown that several of these agents can penetrate post-mortem tissue specimens and significantly shorten the T_1_ and T_2_ relaxation times of mouse brain tissue to enhance signals and tissue contrasts (Sharief and Johnson, 2006). Petiet et al. studied the relationships between tissue T_1_ and T_2_ and the concentrations of ProHance, a Gd-based contrast agent, in the embryonic mouse brain, and showed that tissue T_1_ and T_2_ values change with both the concentration of ProHance as well as the immersion time (Petiet et al., 2007; Petiet and Johnson, 2010). Similar studies have been performed with Gd-DTPA in the mouse brain (Huang et al., 2009; Kim et al., 2009; Cleary et al., 2011), with the T_1_ and T_2_^*^ of the embryonic mouse brain shortened from ~2000 and 40 ms without Gd-DTPA to 50 and 5 ms, respectively, after immersion in 8 mM of Gd-DTPA (Norris et al., 2013a). Interestingly, the same study also demonstrated that Gd-DTPA and a Manganese (Mn)-based contrasts agent (Mn-DPDP) enhanced different gray matter regions in the embryonic mouse brain (e.g., Figure 2 in Norris et al., 2013a). Even though the mechanisms of this selective enhancement are still not well understood, it is a promising approach to enhance tissue contrasts in the *ex vivo* embryonic and neonatal mouse brain MRI. With imaging protocols optimized for resolution and tissue contrasts, T_1_/T_2_-weighted images can now be acquired at spatial resolutions that approach the size of large cells in the brain. For example, Petiet et al. recently reported whole body MRI of embryonic and postnatal mice at a spatial resolution of 0.019 mm and used the technique to characterize anatomical phenotypes in a mutant mouse strain (Petiet et al., 2008; See Table 1 for the imaging parameters).

**Table 1:** **Imaging parameters of selected reports on *ex vivo* MRI of the embryonic and neonatal mouse brain**.

**Study**	**Age**	**Instrument**	**Contrast agent (Concentration)**	**Resolution**	**Contrast and Sequence**	**Scan time**
Petiet et al., 2008	E10.5–E19.5 P0–P32	9.4 T and 7 T	Prohance (20 mM)	0.0195 × 0.0195 × 0.0195 mm	T_1_, 3D SE TE/TR = 5.2/75 ms	3–12 h
Aggarwal et al., 2015	E12.5–E18.5	11.7 T	Magnevist (3 mM)	0.052 × 0.052 × 0.052 mm	3D dw- GRASE, b = 1400 s/mm^2^, 18 directions	21.5–32 h
Norris et al., 2015	E15.5	9.4 T	Magnevist (2 mM)	0.075 × 0.075 × 0.075 mm	3D dw- FSE/SE, b = 1498 s/mm^2^, 42 directions	76 h for 3D SE 19 h for 3D FSE

*dw, diffusion-weighted; FSE, fast spin echo; GRASE, gradient and spin echo; SE, spin echo; T, Telsa; TE, echo time; TR, repetition time*.

### *Ex vivo* diffusion MRI of the developing mouse brain

While conventional T_1_/T_2_-weighted MRI provides satisfactory contrasts for delineation of internal organs and major brain compartments, such as the ventricles and cerebellum, it often lacks good contrasts to further distinguish internal structures within major brain compartments (Zhang et al., 2005), e.g., early white matter tracts. As shown in Zhang et al. (2005), the T_2_ contrasts between gray and white matter structures in the developing mouse brain change dramatically as the brain matures. For example, in embryonic and neonatal mouse brains, the corpus callosum has higher T_2_ values than the surrounding gray matter structures, and this contrast is inverted in the adult mouse brain, which may reflect the changes in tissue water and myelin contents during development and maturation. The lack of consistent contrasts makes it difficult to reliably trace structural development using conventional T_1_/T_2_-weighted MRI. In comparison, diffusion MRI techniques, e.g., diffusion tensor imaging (DTI; Basser et al., 1994; Basser and Pierpaoli, 1996), provide superior contrasts for delineation of the premature gray and white matter structures. The image contrasts of DTI are sensitive to microscopic organization of tissue microstructures, and the contrast patterns remain relatively stable over the late embryonic and early postnatal periods (Zhang et al., 2005). This is one of the advantages that makes diffusion MRI the ideal tool to examine the development of early white matter tracts in the embryonic mouse brain as well as several gray matter structures, e.g., the embryonic cortex (Zhang et al., 2003).

**Figure 1:**
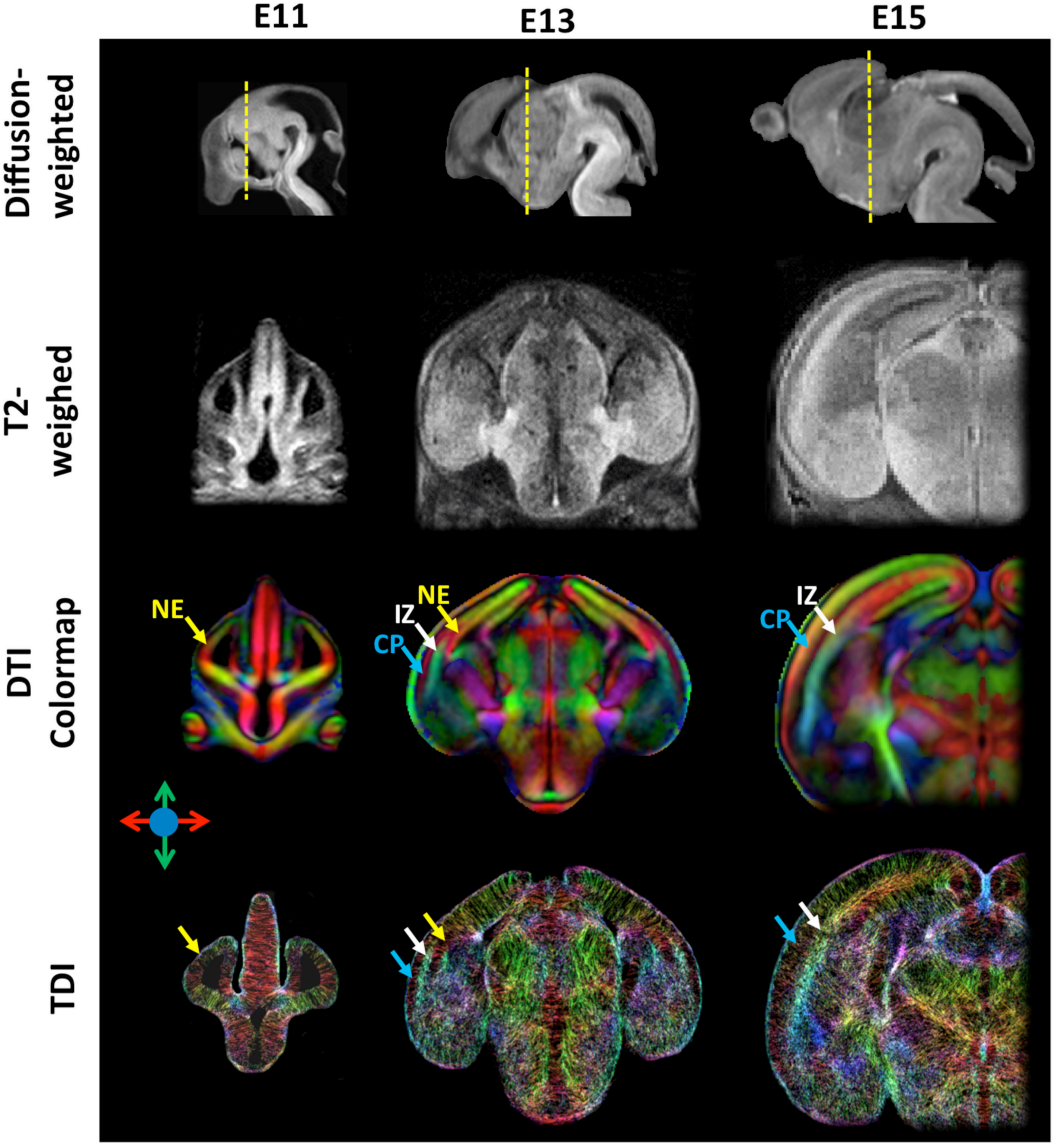
**High-resolution diffusion-weighted, T_2_-weighted, diffusion tensor directionally encoded colormap (DTI colormap), and track density images of embryonic mouse brains at embryonic day 11, 13, and 15**. The diffusion-weighted images show the mid-sagittal plane of the mouse brain, and the T_2_-weighted, DTI colormap, and TDI data show a coronal plane as indicated by the yellow dash lines in the corresponding diffusion-weighted images. The MRI data were acquired at an isotropic spatial resolution of 0.03 mm, and the diffusion MRI data were acquired with 30 diffusion encoding directions and *b*-value of 1300–1500 s/mm^2^. Early cortical structures can be appreciated in the DTI colormaps and TDI data. Structural abbreviations are: CP, cortical plate; IZ, intermediate zone; NE, neuroepithelium. The color scheme used in the DTI colormaps and TDI are: red: left-right; green: ventral-dorsal; blue: rostral-caudal, as indicated by the color arrows.

In the recent years, high-resolution diffusion MRI of the developing mouse brain using sophisticated diffusion MRI techniques has been implemented to enhance our ability to resolve microstructural organizations of the developing mouse brain. Previously, it was common to acquire diffusion MRI data along a small set of 6~12 diffusion encoding directions with a moderate diffusion weighting (*b*-value = 1000–1500 s/mm^2^) and an isotropic spatial resolution of 0.1 mm (Mori et al., 2001; Zhang et al., 2003; Wang et al., 2006). Because DTI is inherently limited by the use of Gaussian model for water diffusion, it cannot resolve complex tissue microstructures, which demands more sophisticated diffusion MRI techniques, such as Q-ball (Tuch et al., 2003), high angular resolution diffusion imaging (HARDI; Frank, 2001), and diffusion spectrum imaging (Wedeen et al., 2005). These techniques, however, all require diffusion MRI data acquired along more diffusion directions (also called high angular resolution) than the minimal set required by DTI and with relatively strong diffusion weighting (*b*-value > 2000 s/mm^2^), which will result in prolonged imaging time and reduced SNR. These challenges can be addressed by using Gd-DTPA and fast imaging sequences. It is necessary to note that, for diffusion MRI, Gd-based contrast agents should be added at lower concentration than conventional T_1_/T_2_-weighted MRI (~ 2 mM for Gd-DTPA vs. 20 mM for ProHance as in Petiet et al., 2008) due to the fact that high concentration of Gd-based contrast agent can shorten tissue T_2_ values to the extent that signal attenuation due to spin-spin relaxation during the diffusion encoding time will cancel the gain from shortened T_1_. Norris et al. reported an optimized for *ex vivo* diffusion MRI of the embryonic mouse brain (Norris et al., 2015). Fast imaging can be achieved using the modified diffusion weighted sequence, such as diffusion weighted fast spin echo or gradient-and-spin echo (GRASE) sequences (Aggarwal et al., 2010; Norris et al., 2013a). Using a GRASE sequence, Aggarawl et al. demonstrated diffusion MRI of the embryonic mouse brain at 0.05 mm isotropic resolution and 12 diffusion-encoding directions (Aggarwal et al., 2010; See Table 1 for the imaging parameters). Figure 1 shows diffusion MRI data acquired from embryonic mouse brains using a modified version of the GRASE sequence at 0.03 mm isotropic resolution and 30 diffusion encoding directions (Wu et al., 2014).

With high spatial and angular resolution diffusion MRI data, several techniques can be used to potentially reconstruct early axonal tracts and resolve complex tissue microstructures in the brain. For example, probabilistic tractography (Behrens et al., 2007) based on constrained spherical deconvolution (CSD; Tournier et al., 2007) allow more robust determination of axonal tracts that cross each other (Moldrich et al., 2010), and tract-density imaging (TDI; Calamante et al., 2010, 2012) facilitates more direct visualization of microstructural organization in the embryonic mouse brain based on high-resolution diffusion MRI data. Aggarwal et al. recently used high-resolution TDI to visualize the changing microstructural organizations in the embryonic mouse cortex (Aggarwal et al., 2015), which share comparable patterns to human fetal brain (Xu et al., 2014).

## *In vivo* MRI of the developing mouse brain

Compared to *ex vivo* MRI, *in vivo* MRI of the developing mouse brain faces several challenges. The total imaging time is often limited to less than 2 h as long exposure of anesthesia has detrimental effects on the developing brain (Liang et al., 2010). In addition, the sensitivity of *in vivo* MRI coils is often lower than *ex vivo* MRI due to the increased coil size needed to accomodate the entire head, the air anesthesia setup, and animal monitoring system. Furthermore, it is often difficult to restrain motions in neonatal mice and mouse embryos in the uterus even with anesthesia. For example, the ear canal and teeth are not fully developed in neonatal mice, and conventional motion restriction setups based on ear pins and bite bars for adult mouse cannot be applied. Due to these challenges, *in vivo* MRI of the embryonic and neonatal mouse brain has not been frequently reported. This has started to change in recent years, and in this section, we will highlight a few recent developments on *in vivo* MRI of the embryonic mouse brain.

### *In vivo* MRI of the embryonic mouse brain

**Figure 2:**
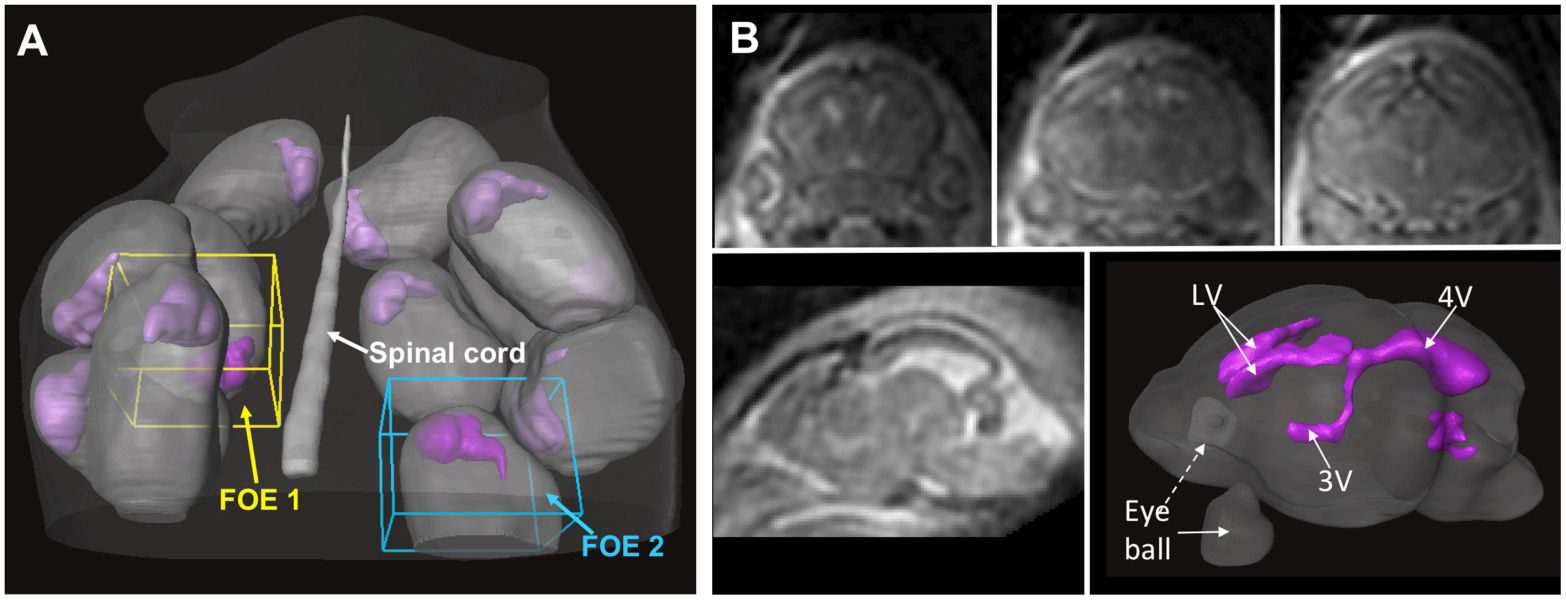
***In utero* T_2_-weighted MRI of an embryonic mouse brain using the localized imaging approach. (A)** Distributions of embryonic sacs (gray structures) and embryonic mouse brains (purple structures) in a pregnant mouse based on whole abdomen multi-slice T_2_-weighted images. Two localized fields of excitation (FOEs) are defined in the whole-abdomen image. **(B)** Coronal (top row) and sagittal (bottom left) T_2_-weighted images of a mouse embryo acquired from one of the localized FOEs. The imaging resolution is 0.12 mm isotropic. The ventricular system (purple structures in the bottom right image) in the embryonic mouse brain can be reconstructed based on the T_2_-weighted images. The images in this figure are modified from Wu et al. (2015) with permission.

*In vivo* MRI of the embryonic mouse brain is extremely challenging. There are usually 6–12 embryos in the uterus of a pregnant mouse, as shown in Figure 2, each within its own embryonic sac. Motions from both the embryos and maternal mice are cause severe motion artifacts, and the large variations in the locations and orientations of embryos make it difficult to apply high-sensitivity surface coils, which limits the SNR. In addition, high resolution in all three dimensions is often required to resolve structures within the miniature brains (< 6 mm in any dimensions). *In vivo* MRI of mouse embryos have been demonstrated before (Hogers et al., 2000; Chapon et al., 2002, 2005) with 2D multi-slice imaging. Recently, Turnbull and colleagues demonstrated successful *in vivo* embryonic mouse brain T_1_-weighted MRI using advanced motion correction techniques on a 7 Tesla MRI system (Nieman et al., 2009; Berrios-Otero et al., 2012; Parasoglou et al., 2013), and have successfully imaged vasculature of embryonic mouse brain and use it to study a mutant mouse strain (Berrios-Otero et al., 2009, 2012). Furthermore, they recently demonstrated that Mn-enhanced MRI (MEMRI) of the embryonic (Deans et al., 2008) and neonatal mouse brain (Szulc et al., 2015; See Table 2 for the imaging parameters used in related studies). They showed that, at various stages, the spatial pattern of Mn enhancement gradually changes, corresponding to neuronal development in the brain. Deans et al. reported that mouse embryos as early as E11.5 can survive for at least 24 h after a single dose of MnCl_2_ of 80 mg/Kg (Deans et al., 2008), suggesting the feasibility of using MEMRI to study embryonic mouse brain development The neurotoxicity of Mn (Sánchez et al., 1993), however, is a potential limiting factor for longitudinal studies. For example, Szulc et al. reported reduced body weights in neonatal mice after Mn exposure (Szulc et al., 2015). Future studies are needed to define the effects Mn exposure on mouse brain development.

**Table 2:** **Imaging parameters of selected reports on *in vivo* MRI of the embryonic and neonatal mouse brain**.

**Study**	**Age**	**Instrument**	**Contrast agent (Concentration)**	**Resolution**	**Contrast and Sequence**	**Scan time**
Szulc et al., 2015	P1–P11	7 T	MnCl_2_ (50 mg/kg maternal i.p. injection, 24 h prior MRI)	0.1 × 0.1 × 0.1 mm	T1, 3D GE TE/TR = 3.6/50 ms	~ 2 h
Deans et al., 2008	E12.5–E18.5	7 T	MnCl_2_ (up to 80 mg/kg maternal i.p. injection, 24 h prior MRI)	0.1 × 0.1 × 0.1 mm	T1, 3D GE TE/TR = 5/40 ms	70–90 min
Wu et al., 2015	E17	11.7 T	Magnevist (0.4 mMol/Kg, maternal i.p. injection, 2 h prior MRI)	0.16 × 0.16 × 0.16 mm	3D dw-GRASE, b = 1000 s/mm^2^, 30 directions	~ 2 h

*dw, diffusion-weighted; FSE, fast spin echo; GRASE, gradient and spin echo; SE, spin echo; T, Telsa; TE, echo time; TR, repetition time*.

**Figure 3:**
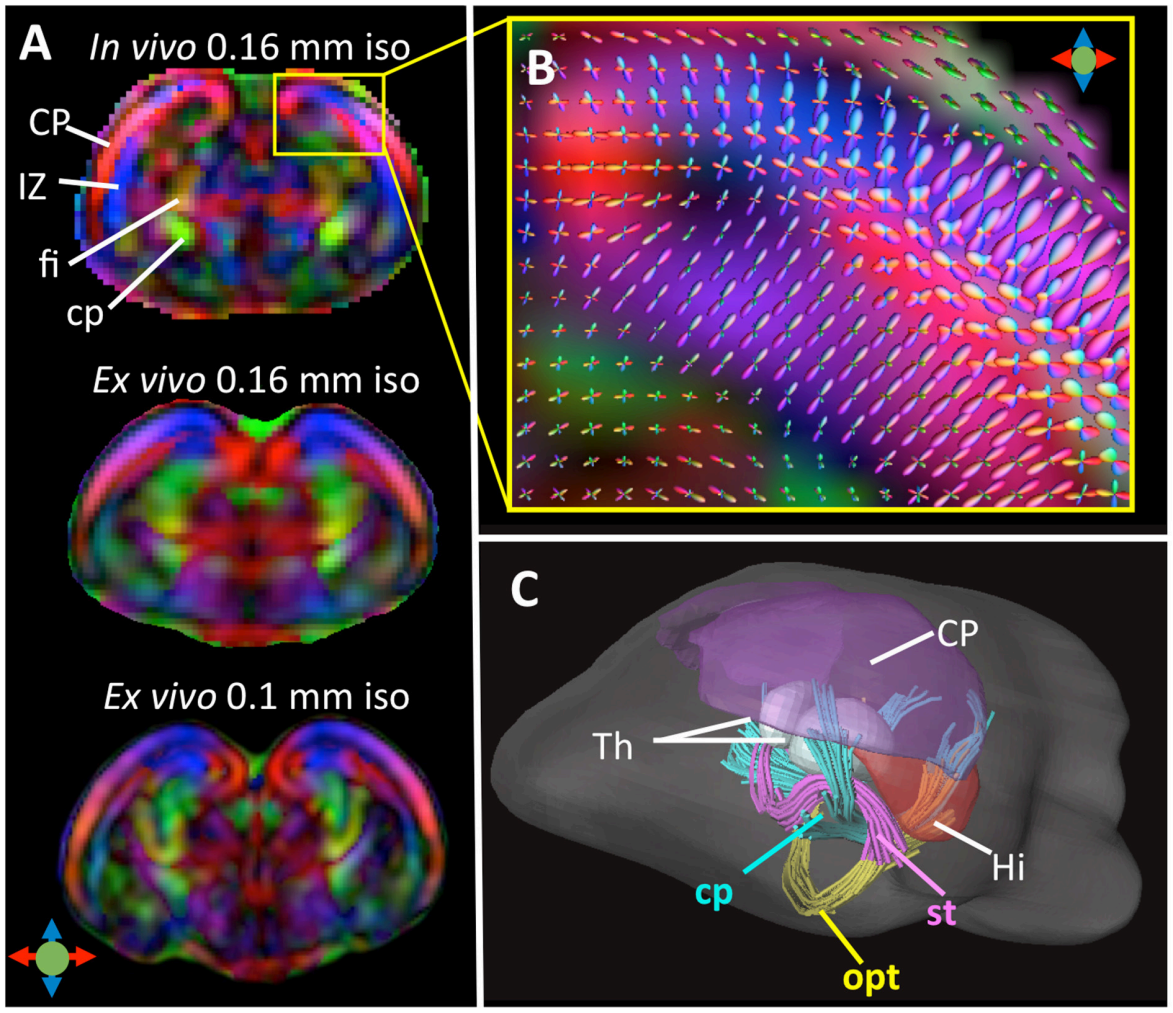
**(A)** Top: A coronal DTI colomap of an embryonic mouse brain at embryonic day 17 (E17) acquired *in vivo* using the localized imaging approach at an isotropic spatial resolution of 0.16 mm. Mid and bottom: comparable *ex vivo* DTI colormaps of an E17 embryonic mouse brain acquired at 0.16 and 0.1 mm isotropic resolution. **(B)** A local fiber orientation distribution (FOD) map showing radially and tangentially distributed microstructures in the cortical plate and intermediate zone, overlapped on a zoomed-in region from **(A)**. **(C)** Early white matter tracts reconstructed from the *in vivo* dMRI data including the cerebral peduncle (cp), optic tract (opt), and stria terminalis (st). Also shown here are several gray matter structures: cortical plate (CP), hippocampus (Hi), and thalamus (Th). The color scheme used in the DTI colormaps and TDI are: red: left-right; blue: ventral-dorsal; green: rostral-caudal, as indicated by the color arrows in **(A)**. The images in this figure are modified from Wu et al. (2015) with permission.

Compared to *in vivo* T_1_/T_2_-weighted MRI, *in vivo* diffusion MRI of the embryonic mouse brain is even more challenging because diffusion MRI is more susceptible to motion, requires length acquisition, and generally has lower SNR due to diffusion-related signal attenuation. In our recent study (Wu et al., 2015), we demonstrated the feasibility of *in-utero* diffusion MRI of the embryonic mouse brain using a localized imaging approach (Finsterbusch, 2010; Schneider et al., 2013) with spatially selective excitation pulses (Pauly et al., 1989; See Table 2 for the imaging parameters). The localized imaging strategy is advantageous because localization can significantly reduce the field-of-view from the whole abdomen to selected embryos, and therefore, shortens the imaging time for 3D imaging and enables high spatial resolution at a given scan time. Combined with a 3D fast imaging sequence and motion correction techniques, we achieved *in-utero* diffusion MRI to study microstructural features in normal and injured embryonic mouse brains. The technique was used to acquire high-resolution T_2_-weighted images (0.12 mm isotropic resolution in 12 mins; Figure 2B) and diffusion MRI data (up to 0.16 mm isotropic resolution, 30 directions in 2 h; Figure 3A). The basic organization of cortical microstructures (Figure 3B) and 3D trajectories of early axonal tracts (Figure 3C), albeit at lower resolution than *ex vivo* MRI results, can be visualized non-invasively for the first time.

## Future directions

In the last decade, multiple technical developments have allowed us to acquire MRI data from the developing mouse brain with increasing resolution. *Ex vivo* MRI can now be routinely used to study anatomical phenotypes in mutant mouse strains. In comparison, *in vivo* MRI, especially, for embryonic mouse brains, still faces many challenges, but has the potential to contribute to better understanding of brain development. Further optimization may improve the resolution and speed of *in vivo* MRI of the embryonic and neonatal mouse brain. New imaging contrasts, e.g., perfusion and more sophisticated diffusion techniques, may be introduced to study the developing mouse brain.

With the increasingly availability of high-resolution MRI data of embryonic and neonatal mouse brains, it is imperative to develop quantitative tools to characterize anatomical phenotypes sensitively and efficiently. MRI-based atlases of the developing mouse brain are important assets (Chuang et al., 2011) to facilitate quantitative measurement of volume and other tissue properties in the brain. Several groups have demonstrated the use of advanced computational tools to characterize the developing mouse brain in terms of their volumetric changes (Verma et al., 2005; Zhang et al., 2005; Baloch et al., 2009) and connectivity (Ingalhalikar et al., 2015). In addition, techniques that can register MRI and histological data have been developed. For example, the Allen brain reference atlas was built on histological images normalized to a MRI dataset of the adult mouse brain (Lein et al., 2007). More sophisticated image registration technique can potentially correct distortions in histological images to a certain degree and form a 3D volume, which will enable systematic examination of the spatial patterns of MRI and histological data.

## Author contributions

All authors listed, have made substantial, direct and intellectual contribution to the work, and approved it for publication.

## Funding

The authors are supported by Howard Hughes Medical Institute (HHMI) International Student Research Fellowship (DW) and National Institute of Health (NIH) NIH R01 NS070909 (JZ) and NIH R01 HD074593 (JZ).

### Conflict of interest statement

The authors declare that the research was conducted in the absence of any commercial or financial relationships that could be construed as a potential conflict of interest.
